# Repeat drug-coated balloon angioplasty for femoropopliteal lesions: 12-month results from a retrospective observational study

**DOI:** 10.1186/s42155-024-00434-w

**Published:** 2024-02-29

**Authors:** Takuya Haraguchi, Masanaga Tsujimoto, Yoshifumi Kashima, Katsuhiko Sato, Tsutomu Fujita

**Affiliations:** Department of Cardiology, Asia Medical Group, Sapporo Heart Center, Sapporo Cardio Vascular Clinic, North 49, East 16, 8-1, Higashi Ward, Sapporo City, Hokkaido 007-0849 Japan

**Keywords:** Drug-coated balloon, Balloon angioplasty, Revascularization, Restenosis, Patency

## Abstract

**Background:**

The clinical implications of restenosis after drug-coated balloon (DCB) treatment remain unclear. We compared the clinical outcomes between DCB angioplasty for restenosis and de novo femoropopliteal artery lesions. This single-center retrospective study included 571 patients (737 limbs) who underwent either repeat (54 patients, 64 limbs) or de novo DCB (517 patients, 673 limbs) without bailout stenting. After propensity score matching, 49 matched pairs were analyzed. The primary endpoint was the 1-year primary patency, with secondary endpoints including the freedom from target lesion revascularization (TLR), major adverse limb events (MALE), and early restenosis. Predictors of restenosis were identified using multivariable Cox regression analysis.

**Results:**

The repeat-DCB group displayed significantly lower rates of 1-year primary patency and freedom from TLR compared to those of the de novo-DCB group (50.1% vs. 77.4%, *p* = 0.029 and 54.9% vs. 83.6%, *p* = 0.0.44, respectively). No significant differences were observed in early restenosis or MALE (10.7% vs. 5.9%, *p* = 0.455 and 48.3% vs. 73.4%, *p* = 0.055, respectively). Restenosis after DCB angioplasty was associated with repeat DCB (hazard ratio [HR], 5.13; 95% confidence interval [CI], 1.43–18.4; *p* = 0.012) and small vessel size of < 4.5 mm (HR, 6.25; 95% CI, 1.17–33.4; *p* = 0.032). Furthermore, restenosis after repeat DCB angioplasty was associated with the Peripheral Artery Calcification Scoring System (PACSS) grade 4 (HR, 4.20; 95% CI, 1.08–16.3; *p* = 0.038), small vessel size of < 4.5 mm (HR, 9.44; 95% CI, 1.21–73.7; *p* = 0.032), and intravascular ultrasound (IVUS) use (HR, 0.05; 95% CI, 0.01–0.44; *p* = 0.007).

**Conclusions:**

The 1-year primary patency rate following repeat DCB angioplasty for femoropopliteal lesions was notably lower than that of DCB treatment for de novo lesions. Repeat DCB strategy was associated with an increased risk of patency loss. Regarding repeat restenosis after DCB treatments, PACSS grade 4 calcification and small vessel diameter of < 4.5 mm were associated with an increased risk of restenosis, whereas IVUS use correlated with a decreased risk of restenosis.

## Background

Conventional endovascular interventions for symptomatic femoropopliteal lesions, such as percutaneous transluminal angioplasty (PTA) with an uncoated balloon or bare metal stent placement, have faced challenges with significant restenosis rates. Recent advancements involving drug-coated balloons (DCBs) and stents offer a potential solution [1–4]. These approaches utilize paclitaxel to inhibit neointimal hyperplasia and smooth muscle cell proliferation, reducing restenosis rates compared to those of traditional PTA. However, consistent reductions in the patency post-DCB angioplasty have been reported across follow-up periods [5, 6].

The Prospective Multicenter Registry of Drug-Coated Balloon for Femoropopliteal Disease study (*n* = 3165) showed a promising patency rate of 84.5% at 12 months and identified prognostic factors for 1-year restenosis after DCB angioplasty [7]. These factors included a history of revascularization, smaller distal reference vessel diameter, severe calcification, chronic total occlusion, low-dose DCB, and residual stenosis. Although increased revascularization procedures correlate with an elevated restenosis risk after DCB, specific treatment strategies remain undisclosed.

A prior study revealed that a 1-year binary restenosis-free status following DCB angioplasty could be better achieved with repeated DCB treatments than conventional PTA (70% vs. 14%, *p* = 0.001) [8]. However, the impact of restenosis after DCB treatment on clinical outcomes remains uncertain. Therefore, this study aimed to assess the clinical outcomes of DCB angioplasty for restenosis after initial DCB treatment and compare them to those of DCB angioplasty for de novo femoropopliteal artery lesions.

## Methods

### Study design and patient population

This retrospective observational study was conducted at Sapporo Heart Center and Sapporo Cardiovascular Clinic in Sapporo, Japan. Between January 2018 and December 2022, 1903 patients with symptomatic lower-extremity artery disease underwent endovascular treatment (EVT). We selectively enrolled 868 patients (1072 limbs) undergoing DCB angioplasty for femoropopliteal lesions with Rutherford classifications of 2–6 [9].

Patients meeting any of the following criteria were excluded: (1) bailout stenting (*n* = 120), (2) common femoral artery lesions (*n* = 87), (3) restenosis after conventional balloon angioplasty (*n* = 59), or (4) in-stent lesions (*n* = 31). The patients were divided into two groups based on whether they underwent DCB angioplasty for new lesions or restenosis after initial DCB angioplasty. The study protocol was approved by the ethics committee of Sapporo Heart Center (No. 20230028) and adhered to the Declaration of Helsinki. The requirement for obtaining written informed consent was waived owing to the retrospective study design.

### Endovascular procedures and medical therapy

Patients with symptomatic femoropopliteal lesions exhibiting stenosis diameters exceeding 70% and a Rutherford classification of 2–6 were identified as EVT candidates. Using a 6 Fr sheath, the common femoral artery was accessed on the appropriate side of the target lesion. An initial dose of 5000 IU of unfractionated heparin was administered, with additional doses administered to achieve an activated coagulation time of at least 250 s. The lesions were crossed using a 0.014- or 0.018-in. guidewire. The balloon size and type were selected based on the lesion morphology and location, using angiographic or intravascular ultrasound (IVUS) data. DCB angioplasty was performed using the operator’s choice of DCB (Lutonix, Becton Dickinson, New Jersey, USA; Ranger, Boston Scientific, Marlborough, USA; and IN.PACT Admiral, Medtronic, Santa Clara, CA, USA) and covered the entire lesion. If angiographic evaluation revealed residual stenosis exceeding 50% following drug-coated balloon (DCB) angioplasty, post-dilatation using an appropriately sized balloon was performed in cases in which bailout stenting was not executed at the operator’s discretion. Atherectomy devices were unavailable during the study period. Hemostasis was achieved via manual compression or closure devices.

Following treatment, a 3-month regimen of dual antiplatelet therapy (daily doses of 100 mg aspirin and 75 mg clopidogrel) was recommended. Patients on anticoagulation therapy received 1 month of combined antiplatelet treatment, followed by a long-term anticoagulation regimen combined with aspirin.

### Study endpoints and follow-up

The primary endpoint of this study was the 1-year primary patency, defined as the sustained openness of the target lesion without significant restenosis. Secondary endpoints included early restenosis within 30 days post-procedure and 1-year clinical events, including the incidence of target lesion revascularization (TLR), overall survival, major amputation, and major adverse limb events (MALE). Restenosis was identified by having either a peak systolic velocity ratio greater than 2.4 on duplex ultrasonography or over 50% stenosis, as evident in follow-up computed tomography angiograms [10, 11]. TLR refers to repeated EVT or surgical revascularization for limbs with recurring symptoms and restenosis. MALE constituted a composite definition and encompassed major amputation, reintervention, or all-cause death [12].

Patient follow-ups were conducted systematically at our outpatient clinic at 1, 6, and 12 months or upon noting clinical deterioration. The protocol involved measuring the postoperative ankle-brachial index and conducting lower-limb duplex ultrasonography during each visit. Additional imaging methods were employed if ultrasonographic results were inconclusive. Lower-extremity artery disease severity was categorized using the Rutherford scale, which distinguished between mild-to-severe intermittent claudication (classes 1–3) and chronic limb-threatening ischemia (CLTI) with or without tissue loss (classes 4–6) [9]. The lesion severity was graded according to the Trans-Atlantic Inter-Society Consensus II classification [13], while the calcification laterality was determined using the Peripheral Artery Calcification Scoring System (PACSS) [14]. The National Heart, Lung, and Blood Institute’s classification system was employed to assess the dissection severity [15].

### Statistical analysis

Continuous variables were expressed as means ± standard deviations and compared using either the unpaired *t*-test or the Mann–Whitney *U* test. Categorical variables, presented as numbers (percentages), were analyzed using the chi-squared test. Propensity score matching was used to reduce intergroup differences in baseline characteristics. The propensity score was calculated using a binary logistic regression model that incorporated the following variables as explanatory factors: coronary artery disease, CLTI, distal reference vessel diameter, hemodialysis, IVUS use, P2Y12 inhibitors, and scoring balloons. The cumulative incidence of study endpoints at 1 year was calculated using the Kaplan–Meier method and compared through the log-rank test. Risk factors for 1-year patency were analyzed using Cox models (chronic total occlusion, CLTI, hemodialysis, high-dose DCB, IVUS use, PACSS grade 4, repeat DCB angioplasty, and a small vessel diameter of < 4.5 mm) [7, 16]. All statistical analyses were performed using SPSS statistics version 29.0 (SPSS, Inc., Chicago, IL, USA). Statistical significance was defined by a two-sided *p*-value < 0.05.

## Results

### Baseline clinical characteristics

**Fig. 1:**
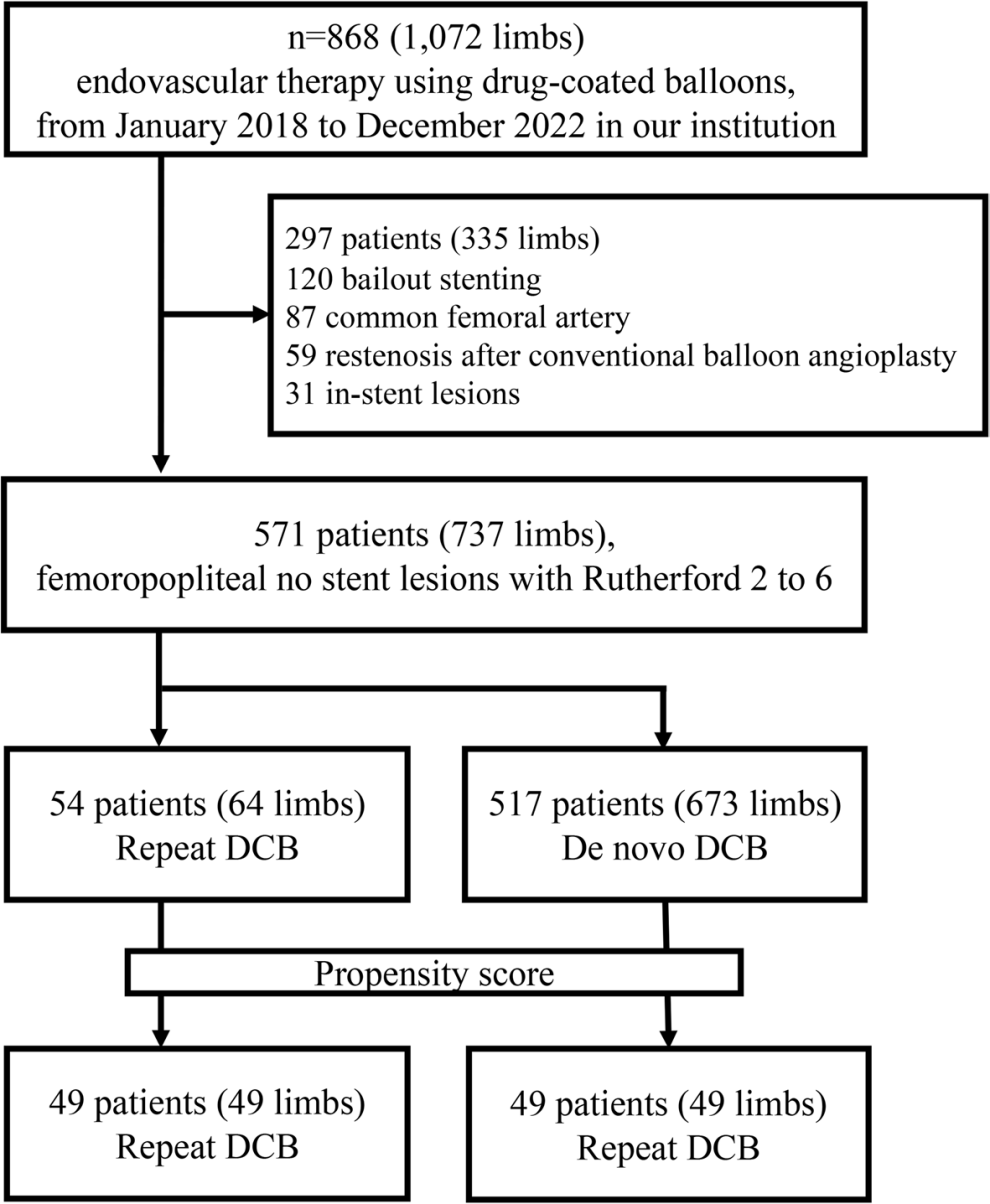
Study flow chart

A total of 571 patients (737 limbs) were enrolled in the present study, with 54 patients (64 limbs) undergoing repeat DCB treatment and 517 patients (673 limbs) receiving de novo DCB treatment. After propensity score matching, 49 matched pairs were analyzed, ensuring comparability in baseline patient and lesion characteristics (Fig. 1 and Table 1). Procedural details, summarized in Table 2, demonstrated no statistical differences between the two groups. Both groups exhibited frequent diffuse lesion morphologies.

**Table 1:** Baseline patient characteristics

	Before matching			Matched population		
	Repeat DCB	De novo DCB	*p*-value	Repeat DCB	De novo DCB	*p*-value
	*n* = 54	*n* = 517		*n* = 49	*n* = 49	
Age, years	75.4 ± 8.1	77.1 ± 9.2	0.19	75.3 ± 7.8	77.4 ± 9.4	0.232
Male sex	57% (31)	58% (302)	0.886	53% (26)	49% (24)	0.686
Body mass index, kg/m^2^	22.4 ± 4.8	22.6 ± 3.8	0.806	21.9 ± 4.6	24.2 ± 4.8	0.027
Ambulatory	70% (37)	71% (366)	0.831	71% (35)	59% (29)	0.203
Current smoker	15% (8)	23% (110)	0.166	10% (5)	19% (8)	0.248
Hypertension	92% (49)	85% (441)	0.161	94% (46)	88% (43)	0.243
Dyslipidemia	72% (38)	65% (336)	0.345	69% (34)	84% (41)	0.095
Diabetes mellitus	57% (31)	52% (268)	0.452	61% (30)	61% (30)	–
Chronic kidney disease	64% (34)	61% (315)	0.671	63% (31)	69% (34)	0.521
Hemodialysis	26% (14)	19% (99)	0.217	26% (13)	24% (12)	0.817
Cerebrovascular disease	17% (9)	19% (98)	0.702	20% (10)	10% (5)	0.161
Coronary artery disease	81% (43)	57% (291)	< 0.001	80% (39)	71% (35)	0.347
Heart failure	17% (9)	15% (75)	0.654	16% (8)	19% (9)	0.754
Aspirin	89% (48)	79% (407)	0.077	92% (45)	86% (42)	0.337
P2Y12 inhibitor	93% (50)	73% (376)	0.001	94% (46)	100% (49)	0.121
Cilostazol	17% (9)	19% (101)	0.611	10% (5)	6% (3)	0.357
Anticoagulant	24% (13)	15% (79)	0.094	20% (10)	20% (10)	–
Statin	61% (33)	51% (264)	0.164	59% (29)	62% (30)	0.738

Data are presented as percentages (numbers) or means±standard deviations, unless otherwise specified

*DCB* drug-coated balloon

**Table 2:** Baseline lesion and procedure characteristics

	Before matching			Matched population		
	Repeat DCB	De novo DCB	*p*-value	Repeat DCB	De novo DCB	*p*-value
	*n* = 64	*n* = 673		*n* = 49	*n* = 49	
Right leg	50% (32)	49% (333)	0.937	47% (23)	49% (24)	0.842
Chronic limb-threatening ischemia	47% (29)	37% (245)	0.095	48% (23)	45% (22)	0.766
Rutherford classification	3.6 ± 0.9	3.5 ± 1.1	0.349	3.6 ± 1.0	3.7 ± 1.3	0.598
Preoperative ankle brachial index	0.71 ± 0.29	0.65 ± 0.30	0.115	0.72 ± 0.25	0.66 ± 0.27	0.256
Postoperative ankle brachial index	0.93 ± 0.17	0.94 ± 0.18	0.548	0.93 ± 0.19	0.93 ± 0.13	0.924
Distal reference vessel diameter (mm)	4.2 ± 1.0	4.6 ± 1.1	0.013	4.1 ± 1.0	4.1 ± 1.0	0.957
Lesion length (mm)	174.9 ± 99.4	187.3 ± 109.5	0.387	170.3 ± 101.2	183.9 ± 124.4	0.556
Preoperative stenosis (%)	90.7 ± 9.7	92.2 ± 9.1	0.214	91.6 ± 9.5	92.0 ± 9.6	0.858
Postoperative stenosis (%)	5.9 ± 12.4	8.6 ± 14.3	0.102	6.6 ± 13.3	4.1 ± 9.3	0.274
Chronic total occlusion	29% (18)	28% (186)	0.874	31% (15)	29% (14)	0.773
Occlusion length (mm)	35.4 ± 72.4	41.2 ± 83.3	0.605	34.0 ± 70.2	37.6 ± 81.2	0.824
Lesion morphology type						
Focal/Tandem/Diffuse	10%/12%/78%	16%/9%/74%	0.415	12%/17%/71%	28%/12%/60%	0.433
Stenosis (Focal/Tandem/Diffuse)	71% (10%/7%/54%)	72% (15%/9%/48%)	0.874	69% (12%/8%/49%)	71% (21%/8%/42%)	0.773
Occlusion (Focal/Tandem/Diffuse)	29% (0%/5%/24%)	28% (1%/3%/24%)	0.874	31% (0%/9%/22%)	29% (7%/4%/18%)	0.773
TASC II A/B/C/D	17%/21%/52%/9%	20%/20%/50%/9%	0.968	19%/19%/54%/8%	27%/17%/46%/10%	0.755
Calcification graded according to the PACSS grade 1/2/3/4	3%/5%/23%/43%	11%/4%/17%/40%	0.214	6%/9%/29%/57%	15%/4%/22%/59%	0.566
Below-the-knee poor-runoff ≤1	47% (29)	37% (245)	0.137	45% (22)	31% (15)	0.166
Drug-coating balloon number	1.4 ± 0.5	1.4 ± 0.5	0.907	1.4 ± 0.5	1.3 ± 0.5	0.679
diameter (mm)	5.6 ± 0.6	5.5 ± 0.6	0.295	5.6 ± 0.6	5.3 ± 0.7	0.093
length (mm)	190.3 ± 91.9	206.2 ± 114.1	0.203	186.9 ± 94.9	191.4 ± 118.2	0.836
Scoring balloon use	57% (32)	42% (271)	0.025	63% (31)	61% (30)	0.835
Intravascular ultrasound use	30% (19)	24% (164)	0.357	31% (15)	18% (9)	0.159

Data are presented as percentages (numbers) or means±standard deviations, unless otherwise specified

*DCB* drug-coated balloon, *PACSS* Peripheral Artery Calcium Scoring System, *TASC* Trans-Atlantic Inter-Society Consensus

### Impact of repeat DCB on clinical outcomes

**Fig. 2:**
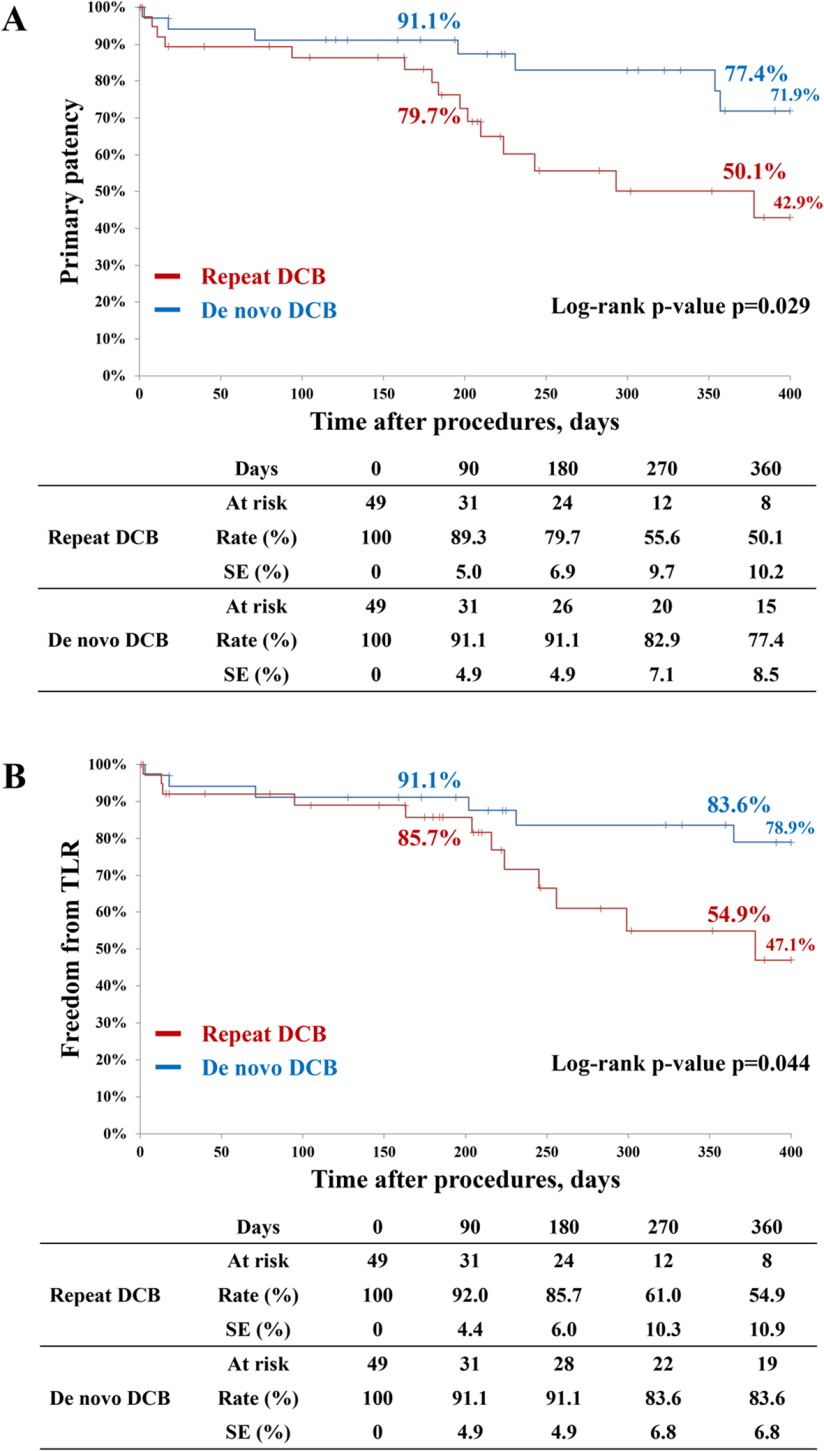
Comparison of (**A**) primary patency and (**B**) freedom from TLR at 1 year between the repeat and de novo DCB groups. DCB = drug-coated balloon, SE = Standard error, TLR = target lesion revascularization

**Fig. 3:**
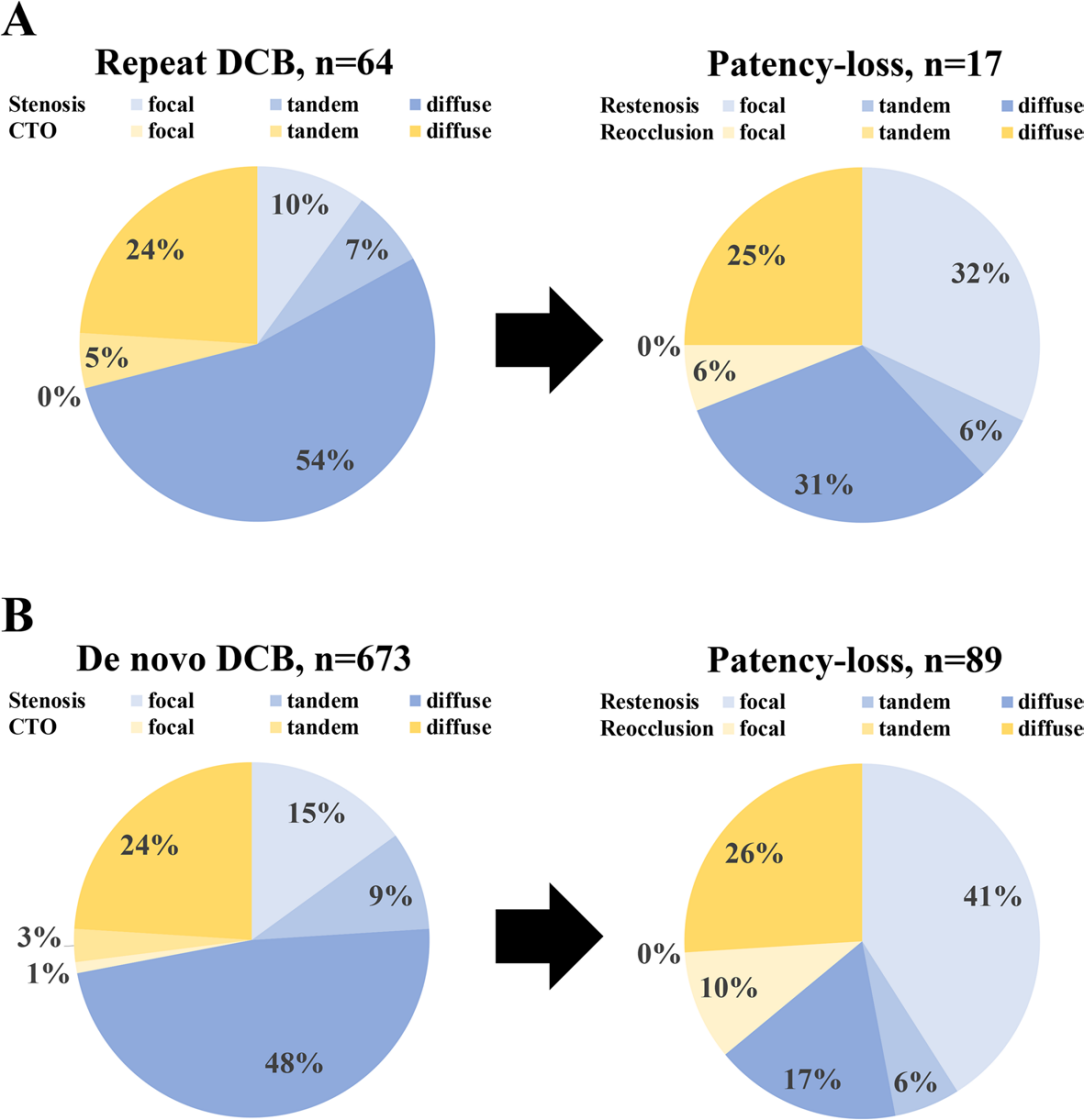
Change in lesion morphology before and after DCB angioplasty in (**a**) the repeat and (**b**) de novo DCB groups

Postprocedural outcomes are presented in Table 3. The primary endpoint of primary patency showed a significant difference, with the repeat-DCB group having lower primary patency than the de novo-DCB group (50.1% vs. 77.4%, *p* = 0.029), as illustrated in Fig. 2a. Freedom from TLR favored the de novo-DCB group (54.9% vs. 83.6%, *p* = 0.044), as shown in Fig. 2b. Rates of early restenosis (10.7% vs. 5.9%, *p* = 0.455), survival (84.6% vs. 80.5%, *p* = 0.848), major amputation (6.4% vs. 3.4%, *p* = 0.543), and MALE (48.3% vs. 73.4%, *p* = 0.055) also showed no significant differences. Lesion morphological patterns of restenosis or reocclusion did not significantly differ between the groups, as demonstrated in Fig. 3a and b.

**Table 3:** Clinical outcomes of endovascular treatment

	Before matching			Matched population		
	Repeat DCB	De novo DCB	*p*-value	Repeat DCB	De novo DCB	*p*-value
	*n* = 64	*n* = 673		*n* = 49	*n* = 49	
Primary patency	56.9%	82.8%	< 0.001	50.1%	77.4%	0.029
Restenosis (Focal/Tandem/Diffuse)	69% (32%/6%/31%)	64% (41%/6%/17%)	0.514	64% (21%/7%/36%)	57% (57%/0%/0%)	0.557
Reocclusion (Focal/Tandem/Diffuse)	31% (6%/0%25%)	36% (10%/0%/26%)	0.514	36% (7%/0%/29%)	43% (0%/0%/43%)	0.557
Freedom from TLR	59.3%	86.0%	< 0.001	54.9%	83.6%	0.044
Major adverse limb event	58.0%	73.9%	0.029	48.3%	73.4%	0.055
Survival rate	86.5%	84.7%	0.943	84.6%	80.5%	0.848
Major amputation	4.6%	2.7%	0.942	6.4%	3.4%	0.543
Early restenosis	8.8%	1.9%	0.009	10.7%	5.9%	0.455
Dissection type A/B/C/D/E	7%/41%/28%/21%/2%	14%/41%/24%/20%/1%	0.268	5%/44%/29%/22%/0%	15%/38%/31%/10%/5%	0.193

*DCB* drug-coated balloon, *TLR* target lesion revascularization

### Factors associated with restenosis

Table 4 presents the interaction analysis for restenosis. In multivariable Cox regression analysis, an increased risk of restenosis was associated with the repeated DCB strategy (hazard ratio [HR], 5.13; 95% confidence interval [CI]: 1.43–18.4, *p* = 0.012) and a small vessel diameter of < 4.5 mm (HR, 6.25; 95% CI: 1.17–33.4, *p* = 0.032). Table 5 shows the clinical factors associated with restenosis in the repeat DCB group. Restenosis after repeat DCB angioplasty was correlated with a PACSS grade of 4 (HR, 4.20; 95% CI: 1.08–16.3, *p* = 0.038), small vessel diameter of < 4.5 mm (HR, 9.44; 95% CI, 1.21–73.7; p = 0.032), and IVUS use (HR, 0.05; 95% CI, 0.01–0.44; *p* = 0.007).

**Table 4:** Independent predictors of restenosis after repeat and de novo DCB angioplasty

	Univariable analysis		Multivariable analysis	
	Hazard ratio (95% CI)	*p*-value	Hazard ratio (95% CI)	*p*-value
Chronic total occlusion	1.09 (0.47–2.56)	0.836	1.52 (0.39–5.89)	0.550
Chronic limb threatening ischemia	3.14 (1.28–7.71)	0.012	2.92 (0.91–9.31)	0.071
Hemodialysis	2.52 (1.01–6.24)	0.046	2.03 (0.54–7.67)	0.295
High-dose DCB	0.77 (0.26–2.29)	0.635	1.27 (0.35–4.64)	0.722
IVUS use	0.46 (0.15–1.36)	0.162	0.22 (0.04–1.12)	0.069
PACSS grade 4	1.51 (0.61–3.72)	0.369	1.31 (0.46–3.78)	0.612
Repeat DCB angioplasty	2.63 (1.07–6.51)	0.036	5.13 (1.43–18.4)	0.012
Small vessel size < 4.5 mm	3.24 (0.76–13.8)	0.113	6.25 (1.17–33.4)	0.032

*CI* confidence interval, *DCB* drug-coating balloon, *IVUS* intravascular ultrasound, *PACSS* Peripheral Artery Calcium Scoring System, *TASC* Trans-Atlantic Inter-Society Consensus Document

**Table 5:** Independent predictors of restenosis after repeat DCB angioplasty

	Univariable analysis		Multivariable analysis	
	Hazard ratio (95% CI)	*p*-value	Hazard ratio (95% CI)	*p*-value
Chronic total occlusion	1.06 (0.38–3.00)	0.091	4.37 (0.62–30.8)	0.139
Chronic limb threatening ischemia	1.84 (0.63–5.34)	0.262	1.13 (0.29–4.40)	0.855
Hemodialysis	2.69 (0.83–8.67)	0.098	6.17 (0.79–48.2)	0.083
High-dose DCB	1.48 (0.46–4.70)	0.514	2.76 (0.58–13.1)	0.201
IVUS use	0.28 (0.08–1.02)	0.053	0.05 (0.01–0.44)	0.007
PACSS grade 4	3.04 (1.04–8.91)	0.042	4.20 (1.08–16.3)	0.038
Small vessel size <4.5 mm	2.33 (0.52–10.3)	0.267	9.44 (1.21–73.7)	0.032

*CI* confidence interval, *DCB* drug-coating balloon, *IVUS* intravascular ultrasound, *PACSS* Peripheral Artery Calcium Scoring System, *TASC* Trans-Atlantic Inter-Society Consensus Document

## Discussion

The key findings of this study can be briefly summarized as follows: (1) the 1-year primary patency and freedom from TLR were significantly lower in the repeat-DCB group than in the de novo-DCB group; (2) comparable incidences of early restenosis, overall survival, major amputation, and MALE were observed between the two groups; (3) the repeat DCB strategy and a small vessel size of < 4.5 mm were associated with an increased risk of 1-year restenosis after DCB angioplasty; and (4) PACSS grade 4 classification and a small vessel size of < 4.5 mm were linked to an increased risk of restenosis, whereas the utilization of IVUS correlated with a reduced risk of restenosis.

Guidelines currently prioritize DCB angioplasty as the initial endovascular approach for femoropopliteal lesions [17, 18]. However, while extensive literature exists on outcomes for in-stent lesions, [19] limited attention has been given to restenosis following DCB treatment. The primary objective of this study was to assess the clinical significance of administering a second dose of paclitaxel. We sought to evaluate the impact of repeated paclitaxel administration by comparing the clinical outcomes of two distinct groups: de novo lesions that were treated with an initial dose of paclitaxel and did not undergo dissection or sustain any other vascular injuries from standard balloon angioplasty, and restenotic lesions that received treatment with a drug-coated balloon (DCB) following an initial DCB angioplasty. The current study illustrates that the repeat DCB strategy yields a lower patency rate, higher revascularization rate, and comparable mortality and major amputation rates compared to de novo DCB treatment for femoropopliteal lesions. Furthermore, the repetition of DCB angioplasty is associated with an increased risk of restenosis in overall DCB treatment, whereas small vessel sizes (< 4.5 mm) are detrimental factors influencing patency. A history of revascularization has been associated with restenosis factors [7]. A history of DCB treatment has also been found to affect the treatment outcomes. Pathological investigations have revealed that restenotic lesions following conventional balloon angioplasty often exhibit increased fibroblast and myofibroblast proliferation, heightened apoptosis, and increased type III collagen content compared to those of de novo lesions [20]. This suggests that patients experiencing restenosis after balloon angioplasty could potentially benefit from drug-based therapies. Furthermore, a comparison of plaque tissue from restenosis after DCB angioplasty with standard balloon angioplasty indicated decreased neointimal thickness and proliferation of smooth muscle cells and fibroblasts, coupled with increased apoptosis and type III collagen content [21]. A previous report demonstrated that repeat DCB treatment improved patency rates compared to conventional balloon angioplasty for restenosis after primary DCB angioplasty, with no discernible change in survival [8]. However, the poor cellularity and increased type III collagen content in restenotic plaque tissue after DCB angioplasty suggests that mechanical scaffolding might offer more effective outcomes than repeat DCB treatment, potentially reducing the risk of recurrent restenosis after DCB. In contrast, the drug-eluting stent has been reported to have a significantly prolonged time to TLR compared to DCB [22]. Based on these insights, it is prudent to consider alternative treatment strategies, such as drug-eluting stents, for addressing restenotic lesions following DCB angioplasty.

The present study demonstrates that PACSS grade 4 calcification and small vessel sizes (< 4.5 mm) are associated with an increased risk of restenosis after repeat DCB angioplasty, whereas IVUS use is associated with a reduced risk. Similarly, previous studies have noted that PACSS grade 4 calcification adversely impacts the patency rate of drug-coated stents and DCBs [7, 23]. Severe calcification pathologically reduces paclitaxel absorption [24]. In severely calcified lesions, DCB angioplasty has demonstrated enhanced patency rates subsequent to the reduction of the calcification volume via atherectomy devices [25]. The use of IVUS in femoropopliteal lesions has been found to reduce restenosis [26]. IVUS aids in precisely characterizing the lesion morphology, vascular features, and minimal lumen area [27], offering more accurate guidance for treatment strategies compared to angiographic methods. Therefore, the integration of IVUS in managing complex lesions, particularly in cases of restenosis following DCB treatment and PACSS grade 4 calcification, is recommended.

**Fig. 4:**
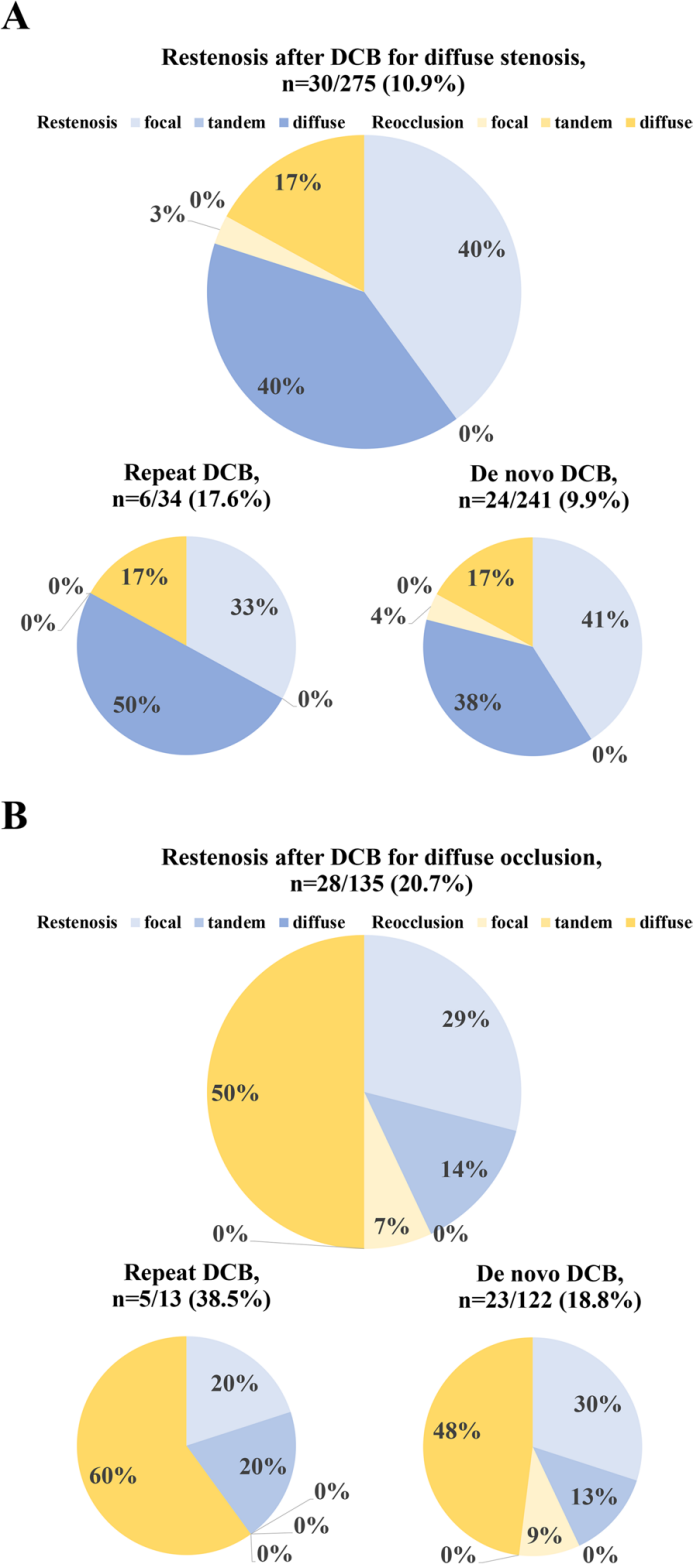
Change in lesion morphology after DCB treatment for (**A**) diffuse stenosis and (**B**) diffuse occlusion. DCB = drug-coated balloon

The restenotic lesion morphology has been characterized by stenosis in 66.1% of cases (focal 37.4%, tandem 9.8%, and diffuse 18.9%) and occlusion in 33.9% [7]. Similarly, the distribution of restenosis patterns after repeat DCB procedures shows that approximately 62% present with stenosis (focal 40%, diffuse 22%), while 38% have occlusion [8]. The present study found no substantial disparity in restenosis morphology patterns between the de novo- and repeat-DCB groups (Table 3), which aligns with the outcomes of previous studies. A previous study has shown that restenosis patterns after drug-coated stent implantation included stenosis in 75% of cases (focal 50% and diffuse 25%) and occlusion in 25%. Diffuse restenosis and occlusion have been associated with worse clinical outcomes than focal restenosis [28]. This study additionally evaluated the restenotic lesion morphology after DCB treatment for diffuse stenosis (*n* = 275) and diffuse occlusion (*n* = 135), as shown in Fig. 4a and b. The restenosis rates for diffuse stenosis and occlusion within the repeat- and de novo-DCB groups were 17.6% (6/34) vs. 9.9% (24/241), respectively (*p* = 0.285) and 38.5% (5/13) vs. 20.5% (23/122), respectively (*p* = 0.140). Diffuse stenosis resulted in restenosis in 10.9% (30/275) of cases. Further exploration of the lesion types associated with restenosis revealed that 80% were characterized as stenosis (focal 40% and diffuse 40%), while 20% displayed occlusion (focal 3% and diffuse 17%). In contrast, diffuse occlusion led to restenosis in 20.7% (28/135) of patients. Upon delving into the restenosis patterns, 43% exhibited stenosis (focal 29% and tandem 14%), and 57% showcased occlusion (focal 7% and diffuse 50%). From these results, the morphology of lesions after DCB treatment for diffuse lesions was less severe in approximately half of the cases than that before DCB angioplasty. This suggests the potential for devising improved treatment strategies that are adaptable to changing lesion morphologies (e.g., diffuse lesions may change to focal restenosis after DCB treatment, allowing focal stenting instead of diffuse stenting in the next treatment); however, prospective studies focusing on changes in lesion morphology after DCB are needed to establish treatment strategies that take into account changes in lesion morphology.

### Limitations

The present study had several limitations. First, this was a retrospective study conducted at a single center. The absence of an independent core laboratory to analyze lesion characteristics and angiographic images could have potentially biased our results and conclusions. Second, while IVUS offers greater accuracy than two-dimensional angiographic imaging, only 24.8% of all patients underwent IVUS, possibly underrepresenting the lesion morphologies. Third, the absence of atherectomy devices during the study period limited the assessment of the impact of severely calcified lesions on clinical outcomes; this assessment could yield different results compared tothe current treatments. Fourth, the present study excluded cases involving bailout stenting for severe dissection and 50% residual stenosis, warranting further studies to compare the clinical outcomes between DCB and stent strategies for restenosis after DCB angioplasty. Finally, a relatively small sample size in the repeat-DCB group may render our findings more hypothesis-generating than definitive. However, the determination of the optimal strategy for addressing restenosis after DCB treatment requires further investigation.

## Conclusions

The 1-year primary patency rate following repeat DCB angioplasty was significantly lower than that observed after de novo DCB angioplasty. Repeat DCB angioplasty and small vessels with diameters < 4.5 mm were associated with a decreased risk of patency after DCB angioplasty. Regarding restenosis after repeat DCB treatment, PACSS grade 4 calcification and small vessel sizes < 4.5 mm were associated with an increased risk of restenosis, whereas IVUS use correlated with a decreased risk of restenosis.

## Data Availability

The data associated with this research are available from the corresponding author upon reasonable request.

## Abbreviations

CLTI: Chronic limb-threatening ischemia
DCB: Drug-coated balloon
EVT: Endovascular treatment
IVUS: Intravascular ultrasound
MALE: Major adverse limb event
PACSS: Peripheral Artery Calcification Scoring System
SD: Spiral dissection
TASC: Trans-Atlantic Inter-Society Consensus
TLR: Target lesion revascularization
